# Factors Increasing the Risk of Recurrence in Fistula-in-ano

**DOI:** 10.7759/cureus.4200

**Published:** 2019-03-07

**Authors:** Nabiyah Bakhtawar, Muhammad Usman

**Affiliations:** 1 Surgery, Lister Hospital, Stevenage, GBR; 2 Internal Medicine, Jinnah Hospital, Lahore, PAK

**Keywords:** anal fistula, fistula-in-ano, fistulotomy, fistula recurrence

## Abstract

Anal fistula, or fistula-in-ano, is a condition involving the anal region that is common yet debilitating. Surgery is the mainstay of treatment for an anal fistula and the chances of recurrence are quite high even after corrective surgical procedures. The risk factors for recurrence can be broadly classified into four categories: 1) risk factors related to the fundamental anatomy of the fistula and presence of comorbidities, 2) lack of proper preoperative assessment of the fistula, which includes failure to recognize the internal opening and overall structure of the fistula and not supplementing the proctologic examination with sufficient imaging, 3) intraoperative loopholes that include improper procedure selection, inexperience of the surgeon, and failure to get rid of the entire tract along with its ramifications, and 4) lack of proper postoperative care in the early and late periods following the surgery.

The aim of this paper, therefore, is to highlight the factors that could increase the risk of recurrence in different types of anal fistulae. Once surgeons know these risk factors, they can anticipate any complication and detect recurrence early.

## Introduction and background

Fistula-in-ano or anal fistula is a common yet debilitating condition involving the anal region. A fistula can be defined as an abnormal connection between two epithelized surfaces lined with granulation tissue. In around 80% of cases, anal fistulae are secondary to a cryptogenic (infectious) process involving the anal glands. Infection of the anal glands leads to abscess formation in the inter-sphincteric planes which can then bud in different directions. Once the tract reaches an epithelized surface, it completes a fistula [1-3]. The incidence of anal fistulae is highly variable. The incidence ranges from as low as 0.7% to as high as 37% in different cases [4-11]. Males are twice as likely to develop an anal fistula compared to females [12]. The mean age of occurrence is 40 years [13-15].

Surgeons have long struggled to find an effective method to treat this cumbersome condition. The treatment of anal fistulae has seen many advancements. Still, the failure and recurrence rates are astoundingly high. The chances of recurrence in different types of anal fistulae range between 7% and 50% [16-18]. Therefore, most patients require multiple surgeries.

Due to a high failure rate in fistula operations and increased risk of recurrence, this condition needs to be studied in detail. Also, there is a need for probing into the factors that increase the risk of recurrence. A clear understanding of these risk factors could help surgeons take timely steps to prevent future complications, especially recurrence. The aim of this paper, therefore, is to shed some light on the factors that increase the risk of recurrence in cases of anal fistulae.

## Review

Factors increasing the risk of recurrence in anal fistulae

The factors increasing the risk of recurrence of an anal fistula can be categorized as:

1. Factors related to the fistula anatomy and other comorbidities

2. Preoperative factors increasing the risk of recurrence

3. Intraoperative deficiencies leading to recurrence

4. Factors related to postoperative complications and care

1. Factors Related to the Fistula Anatomy and Other Comorbidities

Park’s classification is the most useful guide for the diagnosis and categorization of anal fistulae. According to this classification, anal fistulae can be classified into four groups: inter-sphincteric (type I), trans-sphincteric (type II), supra-sphincteric (type III), and extra-sphincteric (type IV). The supra-sphincteric and extra-sphincteric fistulae (type III and IV), though less common, are associated with a higher risk of complications and recurrence [17-19].

A supra-sphincteric fistula starts in the inter-sphincteric plane, continues in the same plane above the puborectalis muscle, and finally moves downward between the puborectalis and levator ani to enter the ischiorectal fossa [19]. In one study, all the patients with a supra-sphincteric fistula developed recurrence. In the same study, supra-sphincteric fistula alone contributed to 39% of cases of recurrence of all the fistula types studied [20].

The extra-sphincteric type is the rarest form of anal fistula. This fistula type has a unique anatomy as it lies completely outside the ring of sphincteric muscles. If this type of fistula is laid open, it would lead to total incontinence, incomplete closure, and consequently will have a higher risk of recurrence [18-19].

Another factor related to fistula anatomy that increases the risk of recurrence is the circumferential involvement of the fistula. This pattern leads to the formation of ‘horse-shoe shaped’ fistula [19]. Koehler et al. in 2004 carried out a research on surgical outcomes in a cohort of patients with horseshoe fistula-in-ano treated with primary closure. According to their experience, 81% of patients underwent multiple procedures. The recurrence rate, following different flap techniques, was as high as 35% [21].

The high extension of a fistula, with erroneous diagnosis and faulty treatment strategy, can also lead to recurrence. All forms of high extending fistulae are difficult to diagnose and manage; some forms are more challenging to deal with than the others. As illustrated by Park et al., inter-sphincteric fistulae with a high track opening into the Iower rectum, high inter-sphincteric fistulae without a perineal opening, and high inter-sphincteric fistulae with a pelvic extension are particularly difficult entities associated with a greater risk of complications and recurrence [19].

Besides the anatomical factors causing recurrent disease, several other comorbidities increase the risk of anal fistula recurrence. These factors include anal cancer [22], Crohn’s disease [23], diabetes [24], smoking [25], and an immunosuppressed state like HIV [26].

2. Preoperative Factors Increasing the Risk of Recurrence

An anal fistula warrants a detailed proctological examination. Performing a proctoscopy is important for several reasons. First, it helps determine the tonus of anal sphincters. Second, it is important to identify the internal opening of the fistula. Finally, a detailed proctoscopy is important to exclude proctitis because of higher risk of recurrence if surgery is performed in the presence of proctitis [27-28].

The internal opening represents the source point of the fistulous pathology. Therefore, effective preoperative and intraoperative identification of the internal opening is crucial for successful treatment and for preventing recurrence. The internal opening of all fistulous tracts should be excised or drained and the wound formed should be left open to heal by secondary intention and to allow proper drainage [29].

The failure to recognize the internal opening preoperatively can lead to a substantial increase in the risk of recurrence. Andrzej et al. reported that the failure to recognize internal opening preoperatively causes a 20-fold increase in the relative risk of fistula recurrence [30]. Therefore, a detailed proctological examination should be supplemented with proper imaging modalities. The success rates of rectal endoscopic ultrasound (EUS) and pelvic MRI in locating internal openings are 86%-95.8% and 90%-96%, respectively [31-32].

A fistula might be complex with multiple tracts and internal openings. At times, it is almost impossible to determine the exact anatomy and relationship of a fistula to the surrounding structures unless proper imaging is available [33-35]. Therefore, lack of proper preoperative imaging might lead to incomplete excision or drainage of the fistulous tract and possible recurrence.

The use of preoperactive rectal EUS is helpful in the case of primary fistulae. In such cases, rectal ultrasound (RUS) helps determine the type of fistula and its internal opening with high accuracy. It is also helpful in the preoperative assessment of anal sphincters. However, EUS is not of much value in cases of recurrent fistulae. That is because EUS is not helpful in differentiating between a scar from a previous fistula and active fistula. Moreover, it gives limited information about the structure of a fistula if the tract is away from the probe [36]. In such conditions, the use of techniques such as injecting hydrogen peroxide and performing MRI and 3D endosonography should be considered [37-39].

3. Intraoperative Factors Leading to Recurrence

The failure to complement clinical examination with proper imaging could increase the risk of recurrence due to some other reasons too. Looking for the internal opening in the absence of proper imaging is quite difficult even for a seasoned surgeon. An attempt to identify the fistulous opening intraoperatively might not be successful because the internal opening might be blocked due to the inflammation of perianal crypts. The use of hydrogen peroxide or a dye to look for the internal opening might also not be successful in such cases. Furthermore, an attempt to use a probe might complicate the situation further. While the gentle use of a probe could help identify the opening, forceful probing could lead to the formation of a false tract, which may even turn into a new fistula on its own (iatrogenic fistula) [40].

Several factors related to the experience of the surgeon performing the procedure are also important. It is important that the surgeon ensures complete course of the fistulous tract, including its ramifications. A failure to recognize and excise the complete fistulous tract can leave behind granulation tissue, which will lead to the persistence of the fistulous tract. Sangwan et al. studied the causes of recurrence in patients with ‘simple’ fistula-in-ano. Complex, fistulae due to Crohn’s, supra-sphincteric, and extra-sphincteric fistulae were excluded from the study. Researchers found that at least 20% of cases of recurrence occurred due to a failure to excise the fistulous tract completely [41].

The problem of recurrence also arises when the surgeon puts aside the basic principles of proctological surgery and tries to emphasize on the cosmetic effects. If a surgeon follows the proctological principles, no matter how extensive the incision, the cosmetic results are quite satisfactory after healing. Therefore, the thought of cosmetic effects should not hold a surgeon back from properly excising or draining a fistula [42].

Moreover, the choice of surgical procedure is one of the most important intraoperative factors controlling the risk of recurrence. The details are summarized in Table 1.

**Table 1:** A table summarizing the risk of recurrence in anal fistulae with different surgical approaches

Procedure	Rate of Recurrence
Fistulectomy	9.5% [43]
Simple fistulotomy	12.5% [43]
Cutting seton fistulotomy	5% to 29% [44]
Fibrin sealants	69% [45]
Anal fistula plugs	32% [46]
Flap procedures	30% to 60% [47]

4. Factors Related to Postoperative Complications and Care

The factors related to the postoperative care of the patients undergoing a fistula surgery have received limited attention. There is limited data that address the issues related to the potential postoperative complications and factors related to postoperative care that could contribute to the risk of recurrence of anal fistulae.

The following points related to the postoperative care of anal fistula treatment have been gathered through a literature search. Lack of these elements of care could potentially lead to an increased risk of recurrence [42,48-50].

Early care (up to four to six weeks): Once operated, the anal canal takes at least six weeks to heal properly. During this period, a thorough medical examination should be performed at least once weekly to rule out potential complications. One of the main complications can be gas and/or fecal incontinence. A thorough examination should be done and the patient should be advised sphincter exercises [48-50].

The wound following anal canal surgery should be irrigated daily and dressing should be changed daily to avoid complications and recurrence. If a ‘cutting’ seton is applied, the patient should be instructed to start pulling on the thread from the fourth week after the surgery and if the doctor has cut the sphincter muscles, the doctor himself/herself will do that four to five weeks after the surgery [42,48-50].

The ‘loose’ setons can stay for a prolonged period. This increases the risk of formation of a fistulous tract around the seton itself. Therefore, care should be taken to irrigate the wound frequently to allow the healing of wound from the base and not from the skin [42,48-50].

Late care (after six weeks): A complete proctological examination should be carried out once every four weeks after the first six weeks of the procedure. This is to help detect early recurrence. A complete proctological examination is of utmost importance and it should be supplemented with a rectal ultrasound [42,48-50].

Complicated fistulae, secondary to Crohn's disease or those that are high lying, will need seton placement for longer duration. The assessment and care for such fistulae should be more frequent and more rigorous [42,48-50].

## Conclusions

To conclude, an anal fistula is a cumbersome condition with a high rate of complications and recurrence. There are a number of risk factors that could possibly increase the chances of recurrence following anal fistula surgery. These risk factors include elements related to the basic anatomy of the fistula, the presence of comorbidities, lack of comprehensive preoperative assessment of the patient, flaws on part of the surgeon, poor choice of operation, and lack of postoperative care. The factors mentioned in this paper should be kept in mind and a surgeon should always anticipate the possible factors in reducing the risk of recurrence.
